# Post-Mating Interactions and Their Effects on Fitness of Female and Male *Echinothrips americanus* (Thysanoptera: Thripidae), a New Insect Pest in China

**DOI:** 10.1371/journal.pone.0087725

**Published:** 2014-01-29

**Authors:** Xiao-Wei Li, Hong-Xue Jiang, Xiao-Chen Zhang, Anthony M. Shelton, Ji-Nian Feng

**Affiliations:** 1 Key Laboratory of Plant Protection Resources and Pest Management, Ministry of Education, Northwest A&F University, Yangling, Shaanxi, China; 2 Department of Entomology, Cornell University, New York State Agricultural Experiment Station, Geneva, New York, United States of America; CNRS, France

## Abstract

Post-mating, sexual interactions of opposite sexes differ considerably in different organisms. Post-mating interactions such as re-mating behavior and male harassment can affect the fitness of both sexes. *Echinothrips americanus* is a new insect pest in Mainland China, and little is known about its post-mating interactions. In this study, we observed re-mating frequency and male harassment frequency and their effects on fitness parameters and offspring sex ratios of *E. americanus* females. Furthermore, we tested the impact of mating and post-mating interactions on fitness parameters of males. Our results revealed that the re-mating frequency in female adults was extremely low during a 30-day period. However, post-mating interactions between females and males, consisting mainly of male harassment and female resistance, did occur and significantly reduced female longevity and fecundity. Interestingly, increased access to males did not affect the ratio of female offspring. For males, mating dramatically reduced their longevity. However, post-mating interactions with females had no effects on the longevity of mated males. These results enrich our basic knowledge about female and male mating and post-mating behaviors in this species and provide important information about factors that may influence population regulation of this important pest species.

## Introduction

During interactions between sexes of an organism, the reproductive interests of males and females may differ and this may result in sexual conflicts [1], [2]. A common sexual conflict in insects involves mating decisions in which males and females may have different preferences, resulting in altered mating rates [2]. It is generally assumed that males benefit by multiple matings which may lead to harassment of females for more mating opportunities [3]. On the other hand, a single or a few matings may provide females with sufficient sperm to reach their reproductive potential [4], [5] and increased mating frequency may have costs to females [6]. Consequently, females often favor a lower mating rate and are resistant or reluctant to re-mate [7].

There is well-documented evidence that repeated mating attempts by males can lead to harassment and female resistance to such harassment can result in substantial costs to females [1]. These costs include physical injuries to the female [8], [9], increased predation [10], reduced female foraging efficiency [11], [12] and energy expenditure [13]. Male harassment can also disturb females during oviposition, reducing their overall fitness [14], [15], longevity [16] and fecundity [17], [18], [19]. Repeated mating attempts by males may also have costs to males [20], although these have not been well studied.

Mating frequency of females varies considerably among different insect species [3]. In some insect species, females mate just once during their lifetime, while numerous insect species mate multiple times, either with different males or with the same male [3],[5],[21]. If a single or a few matings are sufficient for females to fertilize their eggs, there is little need for additional matings and, in some insect species, sexual receptivity is reduced temporarily or permanently in the females after mating [1]. In contrast, in other species, mated females are known to benefit from re-mating and obtaining additional sperm [5] that may reduce any genetic incompatibility between individual males and females [22]. Additionally, males can transfer nuptial gifts to females during mating and these nutrients can enhance a female's fitness [23], [24], [25].

Re-mating frequency of female Thysanoptera varies greatly among different species. In tubuliferan thrips, *Hoplothrips pedicularius, Elaphrothrips tuberculatus* and *H. karnyi*, females can mate repeatedly with the same males before oviposition [26], [27], [28]. Multiple mating of females by different males was also found in tubuliferan thrips *Dunatothrips aneurae* [29]. However, females of some tubuliferan gall-forming thrips mate once and refuse further matings [30]. Refusing additional mating by females has also been reported in some terebrantian thrips [31], [32]. For example, it was reported that after initial mating, *Frankliniella occidentalis* females refused males for more than 5 days [31].

*Echinothrips americanus* is a new thrips species in Mainland China [33], and poses a severe threat to ornamental and greenhouse crops. Like many thrips species, *E. americanus* is arrhenotokous, in which fertilized eggs develop into females and unfertilized eggs develop into males [34]. Thus, studying the progeny sex ratios of mated females can provide insights into sperm utilization and mating strategies in this species. In our previous study, we found that mated *E. americanus* females had higher fecundity and longevity than virgin females [35]. However, in this species there is no detailed knowledge about post-mating interactions such as re-mating behaviors and mating frequency. Direct observation of re-mating behaviors and mating frequency can provide important information about post-mating interactions between females and males and increase our understanding of the basic reproductive biology of this pest. Such knowledge should prove useful for understanding its population dynamics and in the development of management strategies for this new pest in China.

The objective of the present study was to acquire knowledge about post-mating interactions, including female re-mating behavior and mating frequency, and male harassment frequency of *E. americanus*. In addition, we studied the effects of these post-mating interactions on reproduction and survival of female *E. americanus* as well as offspring sex ratios changes in mated females. Furthermore, we tested the impact of reproductive activities on fitness parameters and survivorship of males.

## Results

### Female re-mating frequency and male harassment rate

During the 2 h recording periods, all 20 females tested finished their first mating. The average pre-copulation duration was 1349.4±281.2 seconds (range from 99 to 5781 seconds). The average copulation duration was 553.6±30.4 seconds (range from 116 to 728 seconds).

After initial mating until age 30 days, except one re-mating at age 3 days in one female, no re-mating occurred, regardless of pairing with virgin males or mated males. However, whenever paired with females, males repeatedly harassed females, while females always resisted male advances by arching and twisting and sometimes flicking their abdomen, making it impossible for males to copulate. When males succeeded in mounting females or inserting their aedeagus, females became irritated and violently flicked males away in ca 10 to 20 seconds, which was too short to complete insemination, and significantly shorter than the average copulation duration of 553.6±30.4 seconds.

**Figure 1:**
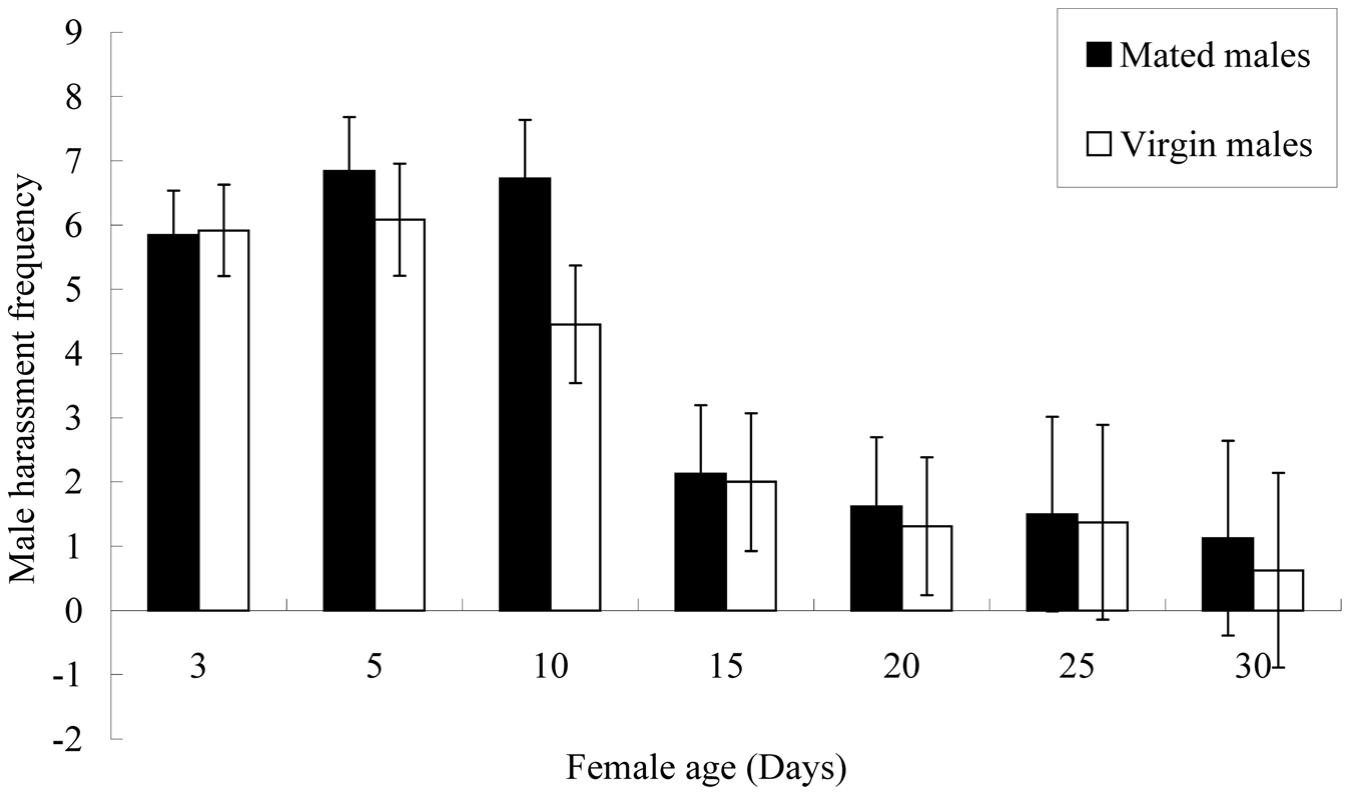
Male harassment frequency by mated and virgin male *Echinothrips americanus* towards females at different ages.

There was no significant difference in male harassment frequency between mated males and virgin males (Table 1). Male harassment frequency significantly decreased with female age (Table 1; Fig. 1). Specifically, male harassment frequency was much higher in the first 10 days, and then decreased dramatically (Fig. 1).

**Table 1:** Effects of *Echinothrips americanus* male mating status and female age on male harassment frequency toward females (two-way ANOVA, *P*<0.05).

Source	df	*F*	*P*
Male mating status	1	0.904	0.344
Female age	6	11.126	<0.0001
Interaction	6	0.390	0.884
Error	118		

### Effects of post-mating interactions on mated female fitness

Mated females without male companions had significantly greater longevity and longer ovipositional periods compared to the other two treatments; however, there was no significant difference in longevity and ovipositional period between females held with one and two males (Table 2). The same results were observed in egg production. Lifetime fecundity of mated females housed with one or two males was significantly reduced compared to females housed alone, while similar levels of egg production were found with one or two males present (Table 2).

**Table 2:** Longevity, oviposition period, fecundity and total female offspring ratios of mated *Echinothrips americanus* females paired with different numbers of males.

	Longevity (days)	Oviposition period (days)	Fecundity (eggs/female)	Total female offspring ratio
1♀×0♂	39.9±2.0a	38.3±2.0a	181.5±9.4a	67.9±3.9a
1♀×1♂	33.6±2.5b	31.7±2.5b	146.2±10.8b	69.9±3.8a
1♀×2♂	31.5±2.1b	29.7±2.1b	132.3±10.7b	76.3±2.8a
*F*; df	3.770; 2, 84	3.977; 2, 84	6.040; 2, 84	1.039; 2, 76
*P*	0.027	0.022	0.004	0.359

Means (± SE) within the same column followed by the same letters are not significantly different at *P*<0.05 level according to one way ANOVA: LSD test. 1♀×0♂: 1 mated female kept alone; 1♀×1♂: 1 female and 1 male kept together until female death; 1♀×2♂: 1 female and 2 males kept together until female death.

**Figure 2:**
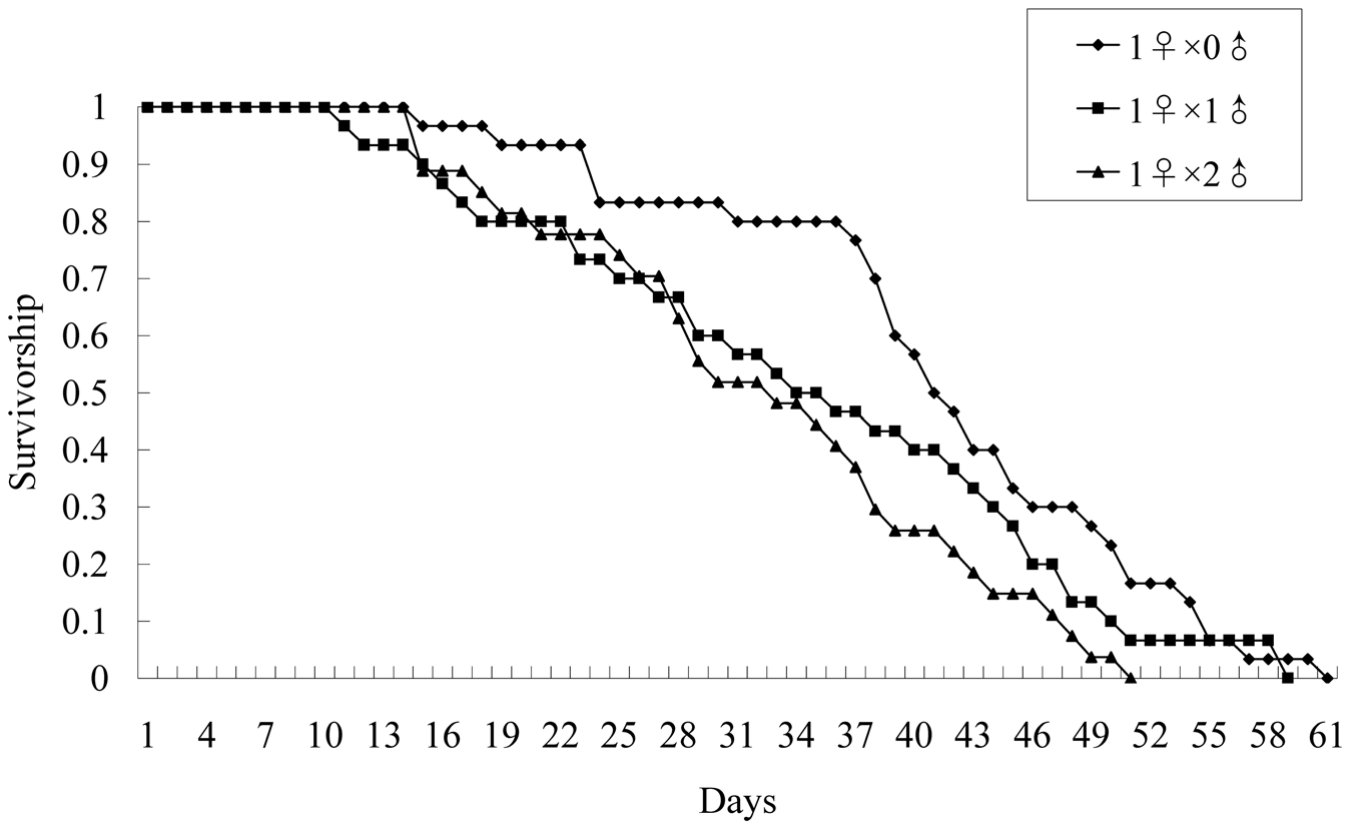
Survivorship of *Echinothrips americanus* mated females paired with different numbers of males. 1♀×0♂: 1 mated female kept alone (*N* = 30); 1♀×1♂: 1 female and 1 male kept together until female death (*N* = 30); 1♀×2♂: 1 female and 2 males kept together until female death (*N* = 27).

Survivorship of mated females was significantly affected by the number of males kept with females (Fig. 2; Log-Rank test: χ^2^_2_ = 6.92; *P* = 0.031). The survival curves of females housed with one male and those housed with two males were not significantly different (Fig. 2; Pair-wise comparison, Log-Rank test: χ^2^_1_ = 1.28; *P* = 0.259). Likewise, the survival curves between females without a male and those housed with one male were not significantly different (Fig. 2; Pair-wise comparison, Log-Rank test: χ^2^_1_ = 1.56; *P* = 0.211). However, there were significant differences in survival between females maintained individually and those housed with two males (Fig. 2; Pair-wise comparison, Log-Rank test: χ^2^_1_ = 8.29; *P* = 0.004). In addition, females held with one male and those held with two males reached their 50% survival rate at nearly the same time (Day 34 and 33, respectively), 7 days earlier than those maintained individually (Fig. 2). These results suggest that interactions with males accelerated female mortality.

### Effects of female age and post-mating interactions on the offspring sex ratios

**Figure 3:**
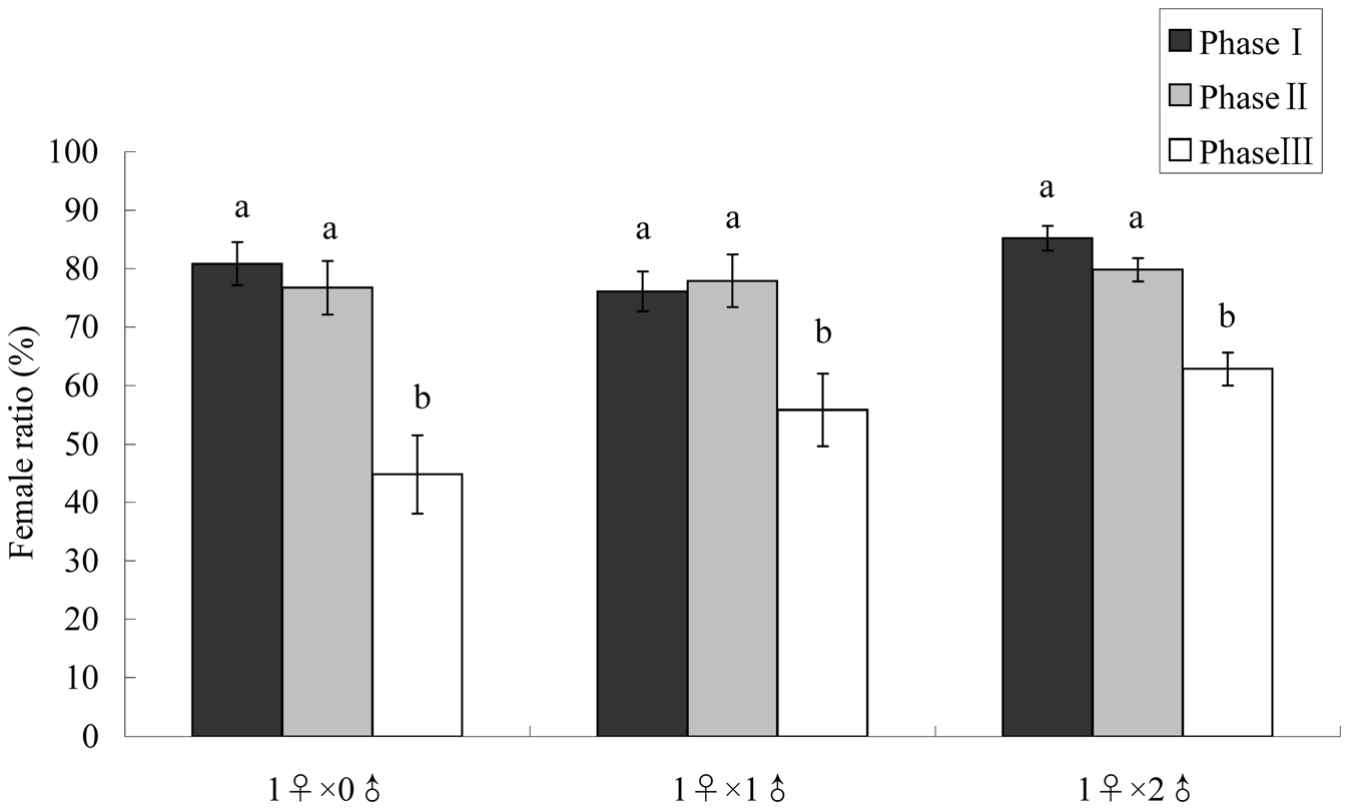
Female offspring ratios of *Echinothrips americanus* females paired with different numbers of males in three phases of their lifespan. Each phase represents one-third of the lifespan. Means within the same treatment followed by the same letters are not significantly different at *P*<0.05 level according to two-way ANOVA: LSD test. 1♀×0♂: 1 mated female kept alone (*N* = 28); 1♀×1♂: 1 female and 1male kept together until female death (*N* = 27); 1♀×2♂: 1 female and 2 males kept together until female death (*N* = 24).

Results showed that female age significantly affected female progeny ratios (Table 3). Specifically, in all three treatments during the first two phases (each phase being a third of its lifespan), there were no significant differences in female ratios; however, during the third phase, fewer female offspring were produced (Table 3; Fig. 3). By contrast, the number of males housed with females had no significant effects on female offspring ratios in each specific phase (Table 3). The same results were observed when comparing total female offspring ratios among treatments (Table 2). In addition, female offspring appeared during the entire lifetime of females in the three treatments, even in the final days of life.

**Table 3:** Effects of number of males paired with females and female age phases (each phase represents one-third of its lifespan) on female offspring ratios of *Echinothrips americanus* (two-way ANOVA, *P*<0.05).

Source	df	*F*	*P*
Male numbers	2	2.388	0.094
Female age phases	2	19.964	<0.0001
Interaction	4	1.212	0.307
Error	228		

### Effects of mating and post-mating interactions on male fitness

**Figure 4:**
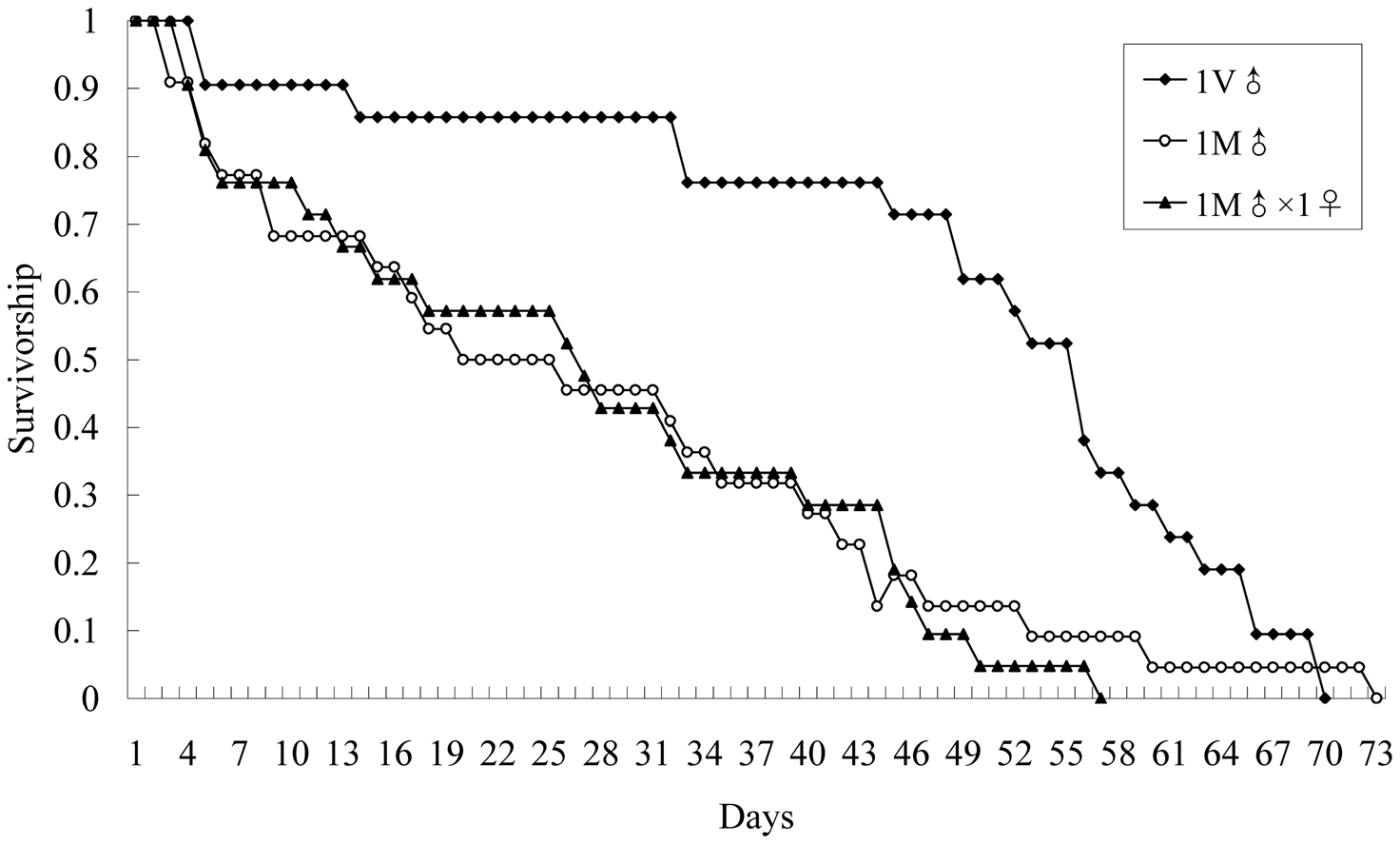
Survivorship of mated *Echinothrips americanus* males in three different treatments. 1V♂: 1 virgin male kept individually (*N* = 21); 1M♂: 1 mated male kept individually (*N* = 22); 1M♂×1♀: 1 mated male kept with 1 female until male death (*N* = 21).

Virgin males lived almost twice as long as mated males (Table 4; Pair-wise comparison, Log-Rank test: χ^2^_1_ = 7.56; *P* = 0.006). In addition, the survival distributions of virgin and mated males were significantly different (Fig. 4; Pair-wise comparison, Log-Rank test: χ^2^_1_ = 7.56; *P* = 0.006). These results suggested that mating behavior was costly for males by reducing their longevity and survivorship. However, for mated males, the presence of females did not significantly affect male longevity and survival (Table 4; Fig. 4; Pair-wise comparison, Log-Rank test: χ^2^_1_ = 0.13; *P* = 0.715).

**Table 4:** Longevity of *Echinothrips americanus* males in 3 different treatments.

Treatments	*N*	Average Longevity (Days)
1V♂	21	48.5±4.3a
1M♂	22	27.0±4.3b
1M♂×1♀	21	26.5±3.8b

Means (± SE) followed by different letters are significantly different (Pair-wise comparison, Log-Rank test, *P*<0.05). 1V♂: 1 virgin male kept individually; 1M♂: 1 mated male kept individually; 1M♂×1♀: 1 mated male kept with 1 female until male death.

## Discussion

This study tested post-mating interactions of female and male *E. americanus* and the influences on female offspring sex ratios, fitness and survival of mated females. We also examined whether mating and post-mating interactions had impacts on male longevity and survival. Our results revealed that the re-mating frequency in female adults was extremely low. During a 30-day period, only 1 of 20 females re-mated once. However, post-mating interactions between females and males, consisting mainly of male harassment and female resistance, did occur and significantly reduced female longevity and fecundity. For the sex ratios, we found the number of males housed with females did not affect the female offspring ratio. Furthermore, the proportion of daughters decreased with female age. For males, mating dramatically reduced their longevity. However, post-mating interactions with females had no effects on the longevity of mated males.

Female mating system are highly diversified in insects and female mating receptivity ranges from females that mate once to those who are always sexually accessible to males [3]. In our study, we found that for up to 30 days, 19 of 20 *E. americanus* females we tested only mated once. After an initial mating, even when males were available and repeatedly attempted to mate with females, females always refused. This refusal of additional matings by females has been found in some other terebrantian thrips [31], [32] and tubuliferan gall-forming thrips [30]. The prevention or reduction of female sexual receptivity to subsequent mating has been reported in many other insect species [1], [36]. This reduction in female receptivity after mating can be due to several factors, including post-copulatory behaviors such as male guarding behavior [37], presence of male ejaculate in the female reproductive tract [38], mating plugs [39] and various re-mating-preventing substances in seminal fluids [40], [41], [42]. In *E. americanus*, post-mating interactions between females and males were primarily male harassment and female resistance; no male guarding behavior was found. So the prevention of re-mating might be caused by the mechanical or chemical factors mentioned above.

When investigating the female progeny ratios of this haplodiploid thrips, we found no changes in the percentage of female offspring among male absent and male present treatments. This suggests that mated *E. americanus* females confined alone did not suffer from sperm limitation at the end of their lifetimes. They received all the sperm they needed from one mating at the beginning and the sperm remained viable in the spermatheca for the majority of their lifetimes. Similar results were reported in Rambur's forktail damselfly, *Ischnura ramburi*, in which there was no sperm limitation in singly mated females [43]. Results from these two species are consistent with the concept that one mating can provide a female with enough sperm to fertilize all of her eggs [4].

Another interesting result was that the proportion of female progeny decreased with female age, although there was sufficient sperm in this species. In some other haplodiploid arthropods, such as wasps and spider mites, even additional matings cannot stop the decline of female progeny production toward the end of their lifetimes [44], [45]. This suggests the decrease in daughter production may be a function of female age [46] rather than sperm insufficiency.

The longevity and fecundity reduction of mated females due to the presence of males has been widely reported in insects including the seed bug, *Lygaeus equestris* [47] and *Callosobruchus* seed beetles [14], [18], [48], [49], although co-habitating with males did not always affect longevity and fecundity at the same time [43], [50], [51]. In our case, extremely low re-mating frequency was found during the majority of the female lifetime and the post-mating interactions were mainly male harassment and female resistance. So the fitness cost observed in our study was due to male harassment and female resistance.

Sexual harassment by males is costly to females. During our experiment, males harassed females 4.4±0.3 times per hour. When males succeeded in mounting females or inserting their aedeagus, females became irritated and violently flicked males away. These resistance behaviors can sometimes cause physical injuries to the female [8], [9]; in addition, the interactions between females and males can increase predation by natural enemies [10], reduce female foraging efficiency [11], [12], increase energy expenditure [13] and disturb oviposition.

There was no difference in female longevity and lifetime fecundity between females housed with one and two males. This indicates male harassment frequency was not linearly related to the number of males. This might be caused by male-male competition for mating opportunities. During male-male competition, each male wastes time and energy, which results in a lower harassment rate compared to single male.

Our results demonstrated that the longevity of mated males was significantly shorter than virgin ones. Costs to males in terms of reduced longevity have also been reported in many other insects, including beetles [52], [53], [54], [55], parasitoid wasps [56] and flies [55], [57]. This longevity cost of mating may be caused by courtship, contest competition, mating or sperm production. In our study, since mated males did not have male companions after mating, longevity reduction may be the result of energy costs during mating as well as ejaculate formation. Surprisingly, there was no difference between mated males living alone and those that stayed with females throughout their lives. This suggests that the reduction of male longevity might primarily occur during the process of mating rather than the interaction with females, and post-mating interactions have no effect on male fitness.

In conclusion, extremely low re-mating frequency and sufficient sperm from a single mating indicates that female *E. americanus* utilize a single mating pattern. Post-mating interactions between females and males are mainly male harassment and female resistance and these interactions exert negative impacts on the fitness of *E. americanus* females but no effects on the sex ratio of their offspring. For males, mating can dramatically reduce their longevity, while post-mating interactions have no effects on male fitness. These results enrich our basic knowledge about the female mating system in this species and provide useful information for population regulation and potential damage by this important pest species.

## Methods

### Population maintenance and insect rearing

The population used in this study was established in late July, 2006 from larvae and adults of *E. americanus* collected from cultivated *Aristolochia ovatifolia* S. M. Hwang in a greenhouse at Northwest A&F University, Yangling, Shaanxi Province, China. For mass rearing of the *E. americanus* population, larvae and adults were kept on potted corn (*Zea mays*) seedlings and maintained in an incubator at 25±1°C, 60±15% relative humidity (RH), and a photoperiod of 14L:10D. To maintain genetic diversity, wild individuals were added to the colony three to four times per year.

For experiments, *E. americanus* in each replication of the treatments were incubated in a sealed glass jar (20 mm diam × 40 mm high) and held at 25±1°C, 60±15% RH, and a photoperiod of 14L:10D. Alfalfa (*Medicago sativa*) leaves were provided as food and an ovipositional substrate and were changed daily.

### Female re-mating frequency and male harassment rate

Twenty 2-day-old virgin females were paired with 2-day-old males and observed using a video recorder for 2 h for mating to occur. Pre-copulation duration and copulation duration were determined. Thereafter, at female ages of 3, 5, 10, 15, 20, 25 and 30 days, each mated female was paired with a young (2–7 d old) mated male and virgin male for 2 h, respectively, and behaviors were recorded using a video recorder. After recording, males were removed and females were left alone until the next recording day. Some females died before the experiment was finished and, thus, the number of females tested per time period decreased with time. Between time periods, females were held in tubes under the rearing conditions described above. Male harassment frequency (male harassment incidences per hour) was also determined. We considered a male harassment incident when a male mounted a female's back and twisted his abdomen sideways under the end of the female's abdomen.

### Effects of post-mating interactions on mated female fitness

Three treatments were established to test the effects of post-mating interactions on the fitness of mated females: Treatment 1) 1 mated female kept alone (*N* = 30); Treatment 2) 1 female and 1 male kept together until the female's death (*N* = 30); Treatment 3) 1 female and 2 males kept together until the female's death (*N* = 27). To start the experiments, pupae of *E. americanus* were individually incubated with alfalfa leaves in glass jars. After emergence, each female adult was paired with one or two young (2–7 d old) males according to the arrangement of treatments above. For treatment 1, one male was kept with each female and then removed after mating, while for the other two treatments, males were left with females during the entire lifespan of the females. In case of the male's death before the female's, new young (2–7 d old) males were added to replace the dead ones so that females were accompanied by the same number of males during their lifetimes.

Alfalfa leaves were checked at 24-h intervals using the bottom light of the stereomicroscope to record the number of eggs produced by each female. Leaves with eggs were replaced with fresh leaves and were moved into new jars for the analysis of sex ratios. Longevity, oviposition period and lifetime fecundity were calculated for each female.

### Effects of female age and post-mating interactions on the offspring sex ratios

Offspring from the three treatments were used to determine the sex ratios. When the eggs hatched and larvae grew into adults, their sex was determined using a stereomicroscope, and the female ratio of offspring each female produced daily was recorded. Longevity of each female was divided into three equal phases (if the lifespan in number of days was not a multiple of 3, the first two phases were equal number of days while the third phase was one day more or less than the other two phases). The female sex ratios of each phase and lifetime female sex ratios in each treatment were calculated.

### Effects of mating and post-mating interactions on male fitness

To test whether mating and post-mating interactions with females affected the lifespan and survival of males, 3 treatments were established: (1) 1V♂: 1 virgin male kept individually, having no access to females (*N* = 21); (2) 1M♂: 1 mated male kept individually (*N* = 22); (3) 1M♂×1♀: 1 mated male kept with 1 female until male death (*N* = 21). Thrips in the treatments were kept in glass jars with alfalfa leaves that were changed daily. For treatment 3, another mated female was paired with males after the female died.

### Statistical analysis

All data analyses were performed in SPSS (v16, SPSS Inc, Chicago, IL, USA). Prior to analysis, data were checked for normality using non-parametric Kolmogorov-Smirnov test (*P*<0.05) and all percentage data were arcsine or square root transformed, as necessary, but untransformed means are presented. The effects of male mating status and female age on male harassment frequency were analyzed by two-way analysis of variance (ANOVA) and least significant difference (LSD) tests (*P*<0.05). For female experiments, the differences in longevity, oviposition period, lifetime fecundity and total female offspring ratios were compared using one-way analysis of variance (ANOVA) and least significant difference (LSD) tests (*P*<0.05). Female offspring ratios in different treatments and different phases were analyzed by two-way analysis of variance (ANOVA) and least significant difference (LSD) tests (*P*<0.05). For survival analysis, Log-Rank test was applied to compare the survival distributions of the female adults.

For male experiments, we used survival analysis (Log-Rank test) to test for differences in lifespan and survivorship among treatments.
